# Supplementary material for: Evaluation of Current Amikacin Dosing Recommendations and Development of an Interactive Nomogram: The Role of Albumin
Source: Pharmaceutics. 2021 Feb 15;13(2):264. doi: 10.3390/pharmaceutics13020264 (PMC7919491; doi:10.3390/pharmaceutics13020264)
Supplement: Supplementary file 1 [file pharmaceutics-13-00264-s001.pdf]

pharmaceutics

MDPI

Supplementary Materials: Evaluation of Current Amikacin Dosing Recommendations and Development of an Interactive Nomogram: The Role of Albumin

Jonás Samuel Pérez-Blanco, Eva María Sáez Fernández, M Victoria Calvo, José M Lanao And Ana Martín-Suárez

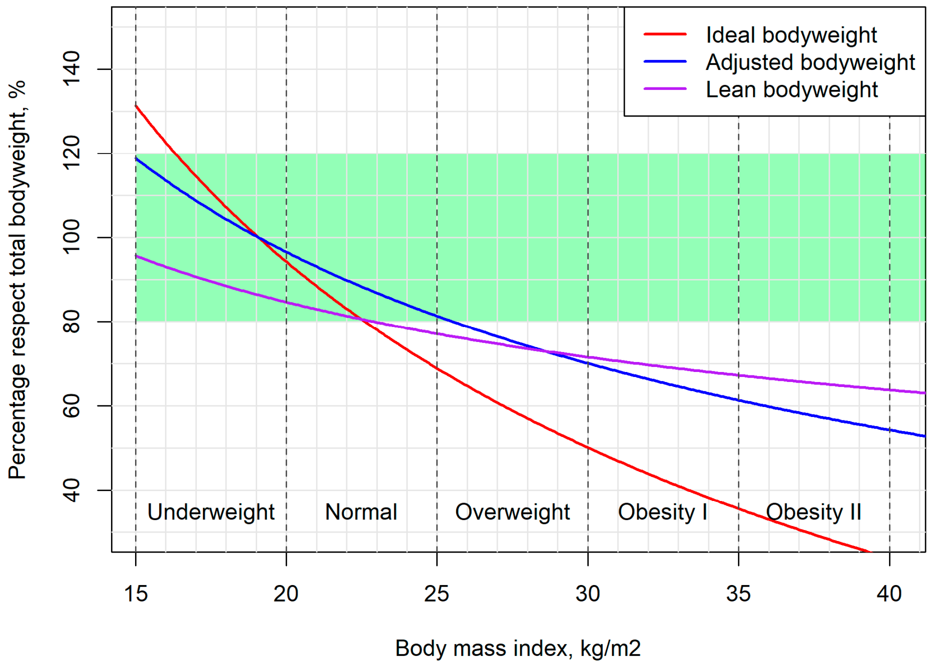

Figure S1. Comparison between different bodyweight measures and total bodyweight (TBW) classified according to body mass index (kg/m^2). Red, blue and purple solid lines, percentage of ideal bodyweight (IBW), adjusted bodyweight (ABW) and lean bodyweight (LBW) respect to TBW, respectively. Green area, situations where weights calculated with the different equations would be within ±20% of TBW. IBW (kg) = 50 kg + [0.9 × (height (cm) − 152.4 cm)] [1] ABW (kg) = IBW (kg) + 0.4 × [TBW (kg) − IBW (kg)] [1] LBW (kg) = [1.1013 × TBW (kg)] − [0.01281 × BMI × TBW (kg)] [2].

Table S1. Summary of amikacin population pharmacokinetic models developed in adults.

Reference	Population				Treatment and Sampling		Covariates in Final Model		Estimated Parameters ¶ (IIV, CV%)				Software
	N (male)	Age (years)*	Albumin (g/dL)*	Patients	Amikacin dose	Samples	CL	Vd	CL (L/h)	V_1 (L)	Q (L/h)	V_2 (L)	
Pérez-Blanco [3]	215 (124)	61 [18–93]	2.9 [1.2–5.0]	General medicine and critically ill	250–2000 mg q24 h	623	eGFR, vancomycin	Albumin, weight	4.78 (28.3)	26.3 (10.4)	-	-	NONMEM
Burdet [4]	60 (47)	61.5 [28–84]	1.9 [1.4–4.4]	Critically ill	11–28 mg/kg q24 h	291	CL_{CR}	PaO_2/FiO_2, weight	4.3 (31)	15.9 (22)	12.1 (27)	21.4 (47)	Monolix
Matar [5]	56 (32)	57.4 [19–90]$^{\Delta}$	-	Critically ill	500 mg q12 h	331	CL_{CR}	-	5.08 (NA)$^{\Psi}$	16.7 (38)	36.9 (NA)$^{\Psi}$	25.8 (NA)$^{\Psi}$	USC PACK
Jang [6]	197 (113)	61.0 ± 17.5	-	General medicine and critically ill	125–1000 mg q24 h	698	CL_{CR}, ward	Cholecystitis, weight	2.8 (30)	18.0 (NA)	-	-	NONMEM
Delattre [7]	88 (57)	65.0 [22–89]	1.8 [0.8–4.9]	Sepsis	25 mg/kg q24 h	507	CL_{CR}	-	0.77 (59)	19.2 (39)	4.4 (17)	9.4 (44)	NONMEM
Lugo-Goytia [8]	42 (NA)	59 ± 15	NA	Sepsis	7.5–30 mg/kg q8–q24 h	NA	Catecholamines, CL_{CR}, PEEP	APACHE II	3.85 (41)	31.7 (29)	-	-	USC PACK
Joubert [9]	14 (NA)	52.7 ± 20.2	-	Critically ill	600–1350 mg q24 h	744	CL_{CR}	-	-	17.1 (22.2)	5.22 (104)	-	NONMEM
Romano [10]	134 (77)	53.0 ± 16.4	-	Haematological	18.5 ± 5.5 mg/kg q24 h$^{\beta}$	3.2 ± 1.9$^{\beta}$	AML diagnosis, CL_{CR}	Hypoalbuminemia, weight	No AML: 5.53 (29) AML: 6.63 (29)	Normoalbumin: 23.98 (26) Hypoalbumin: 31.17 (26)	-	-	NONMEM
Romano [11]	120 (73)	52.9 ± 18.5	-	Critically ill	15.6 ± 6.2 mg/kg q24 h$^{\beta}$	4.2 ± 2.7$^{\beta}$	CL_{CR}, trauma diagnosis	Sepsis diagnosis, weight	No trauma: 4.5 (28.2) Trauma: 5.5 (28.2)	No sepsis: 27.1 (23.2) Sepsis: 33.6 (23.2)	-	-	NONMEM
Tod [12]	57 (35)	51.0 ± 16.0	NA	Haematological	7.5 mg/kg q12 h or 20 mg/kg q24 h	278	Age, creatinine, sex, weight	-	Male: 3.82 (21) Female: 3.40 (21)	8.92 (15)	4.43 (30)	11.4 (25)	NONMEM
Lugo [13]	73 (NA)	60.0 ± 12.0$^{\Omega}$ 57.8 ± 8.0†	-	Sepsis	7.5–30 mg/kg q24 h	NA	Cirrhosis	Cirrhosis	-	No cirrhosis: 31.0 (NA) Cirrhosis: 41.5 (NA)	-	-	USC PACK

Lugo [14]	30 (17)	50.0 ± 15.0	2.4 ± 0.6	Sepsis	7.5 mg/kg◆	NA	Catecholamines, CL_{CR}, PEEP	Albumin, oxygen extraction ratio, weight	3.6 (34)	32.0 (35)	-	-	PCNonlin
Debord [15]	40 (30)	51.8 ± 18.2	-	Critically ill	2.2–31.4 mg/kg q24 h	212	CL_{CR}	-	4.5 (69)	25.6 (28)	-	-	USC PACK
Maire [16]	50 (NA)<br>50 (NA)	62 (±NA)<br>80 (±NA)	-<br>-	General medicine<br>Geriatrics	NA<br>NA	124<br>277	CL_{CR}	Weight	NONMEM: 2.74 (70)<br>NPEM: 3.76 (46)<br>NPML: 3.77 (57)	NONMEM: 19.6 (28)<br>NPEM: 22.3 (33)<br>NPML: 21.4 (36)	-	-	NONMEM<br>USC PACK
Debord [17]	40 (NA)	51 [18–77]Δ	-	Critically ill	7.5 mg/kg q24 h	7 (NA)β	-<br>-	-<br>-	-<br>-	0.4 L/kg (NA)<br>0.36 L/kg (NA)	-<br>0.013 (NA)Ψ L/kg	-<br>1.08 (NA)Ψ L/kg	USC PACK<br>USC PACK

AML, acute myeloblastic leukaemia; APACHE, Acute Physiology and Chronic Health Evaluation; CL, total clearance; CL_{CR}, creatinine clearance; CV, coefficient of variation; eGFR, estimated glomerular filtration rate; IIV, inter-individual variability; NA, not available; PaO_2/FiO2, ratio between the partial pressure of arterial oxygen and the fraction inspired oxygen; PEEP, positive end-expiratory pressure; Q, intercompartmental clearance; Vd, volume of distribution; V_1 or V_2, volume of distribution of central and peripheral compartment, respectively; Weight, total bodyweight expressed in kilograms. ¶ Values expressed as mean. * Values expressed as mean ± standard deviation or median [range]. Δ Values expressed as mean [range]. β◆ Values expressed as mean ± standard deviation per patient. Ω Patients without cirrhosis. † Patients with cirrhosis. ◆ Dose interval adjusted according to pharmacokinetic dosing method. Ψ Parameters calculated with the microconstant values provided in the population pharmacokinetic models: k_{12}, first-order transfer rate constant from the central compartment to the peripheral compartment; k_{21}, first-order transfer rate constant from the peripheral compartment to the central compartment; Kslope, renal component of the elimination rate constant. IIV of each microconstant available in the population pharmacokinetic models.

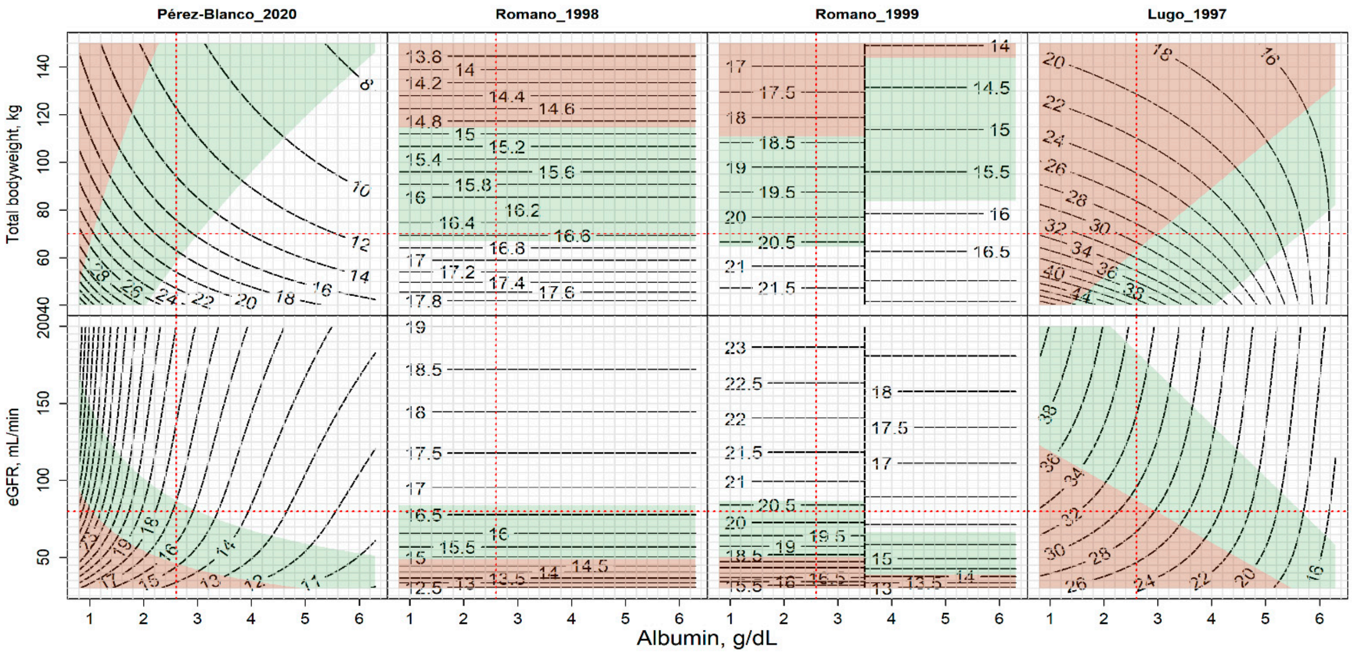

Figure S2. Impact of PopPK model on amikacin dosage required for Cmax/MIC criterion. Amikacin dose (mg/kg) administered once-daily in 1 h infusion required to reach a Cmax/MIC = 10 across albumin, total body weight and estimated glomerular filtration rate (eGFR) variations in the absence of concomitant drugs (vancomycin, catecholamine), ICU male patient without sepsis or trauma, no acute myeloid leukaemia diagnosis, oxygen extraction ratio of 23.5% and positive end-respiratory pressure of 10 cm of H_2O when applicable. eGFR: estimated glomerular filtration rate calculated with Jellife [18], CKD-EPI [19], corrected creatinine clearance for ICU patients' equation [20] and Cockcroft-Gault [21] or Romano_1998 [11], Pérez-Blanco_2020 [3], Lugo_1997 [14] and Romano_1999 [10], respectively. Upper panels, eGFR fixed to 80 mL/min. Bottom panels, total bodyweight fixed to 70 kg; red dashed lines, subject of 70 kg, 2.6 g/dL of albumin and eGFR of 80 mL/min; MIC = 4 mg/L; green and red areas, scenarios where concentrations above MIC ($T_{>MIC}$) represent 60% of the time of dosing interval administration (efficacy) and minimum concentrations of 4 mg/mL (toxicity), respectively.

References

1. Gilbert, D.N.; Chambers, D.N.; Eliopoulos, G.M.; Saag, M.S.; Pavia, A.T.; Black, D.; Freedman, D.O.; Kim, K.; Schwartz, B.S. *The Sanford Guide to Antimicrobial Therapy 2019: 50 Years: 1969-2019*; Editorial Médica A.W.E.E.S.A: Madrid, Spain, 2019; ISBN 978-987-639-059-0.
2. Bonate, P.L. *Pharmacokinetic-Pharmacodynamic Modeling and Simulation*; Springer Science & Business Media: Berlin/Heidelberg, Germany, 2006; ISBN 978-0-387-27199-6.
3. Pérez-Blanco, J.S.; Sáez Fernández, E.M.; Calvo, M.V.; Lanao, J.M.; Martín-Suárez, A. Amikacin initial dosage in patients with hypoalbuminaemia: An interactive tool based on a population pharmacokinetic approach. *J. Antimicrob. Chemother.* **2020**, *75*, 2222–2231, doi:10.1093/jac/dkaa158.
4. Burdet, C.; Pajot, O.; Couffignal, C.; Armand-Lefèvre, L.; Foucrier, A.; Laouénan, C.; Wolff, M.; Massias, L.; Mentré, F. Population pharmacokinetics of single-dose amikacin in critically ill patients with suspected ventilator-associated pneumonia. *Eur. J. Clin. Pharmacol.* **2015**, *71*, 75–83, doi:10.1007/s00228-014-1766-y.
5. Matar, K.M.; Al-lanqawi, Y.; Abdul-Malek, K.; Jelliffe, R. Amikacin population pharmacokinetics in critically ill Kuwaiti patients. *BioMed Res. Int.* **2013**, *2013*, 202818:1–202818:8, doi:10.1155/2013/202818.
6. Jang, S.B.; Lee, Y.J.; Park, M.S.; Song, Y.G.; Kim, J.-H.; Kim, H.K.; Ahn, B.S.; Park, K. Population pharmacokinetics of amikacin in a Korean clinical population. *Int. J. Clin. Pharmacol. Ther.* **2011**, *49*, 371–381, doi:10.5414/cp201520.
7. Delattre, I.K.; Musuamba, F.T.; Nyberg, J.; Taccone, F.S.; Laterre, P.-F.; Verbeeck, R.K.; Jacobs, F.; Wallemacq, P.E. Population pharmacokinetic modeling and optimal sampling strategy for bayesian estimation of amikacin exposure in critically ill septic patients. *Ther. Drug Monit.* **2010**, *32*, 749–756, doi:10.1097/FTD.0b013e3181f675c2.
8. Lugo-Goytia, G.; Castañeda-Hernández, G. Bayesian approach to control of amikacin serum concentrations in critically ill patients with sepsis. *Ann. Pharmacother.* **2000**, *34*, 1389–1394, doi:10.1345/aph.19104.
9. Joubert, P.; Bressolle, F.; Gouby, A.; Douçot, P.Y.; Saissi, G.; Gomeni, R. A population approach to the forecasting of amikacin plasma and urinary levels using a prescribed dosage regimen. *Eur. J. Drug Metab. Pharmacokinet.* **1999**, *24*, 39–46, doi:10.1007/BF03190009.
10. Romano, S.; Fdez de Gatta, M.M.; Calvo, M.V.; Caballero, D.; Dominguez-Gil, A.; Lanao, J.M. Population pharmacokinetics of amikacin in patients with haematological malignancies. *J. Antimicrob. Chemother.* **1999**, *44*, 235–242, doi:10.1093/jac/44.2.235.

11. Romano, S.; Fdez de Gatta, M.D.M.; Calvo, V.; Mendez, E.; Domínguez-Gil, A.; Lanao, J.M. Influence of clinical diagnosis in the population pharmacokinetics of amikacin in intensive care unit patients. *Clin. Drug Investig.* **1998**, *15*, 435–444, doi:10.2165/00044011-199815050-00008.
12. Tod, M.; Lortholary, O.; Seytre, D.; Uzzan, B.; Guillevin, L.; Casassus, P.; Petitjean, O. Population pharmacokinetic study of amikacin administered once or twice daily to febrile, severely neutropenic adults. *Antimicrob. Agents Chemother.* **1998**, *42*, 849–856, doi:10.1128/AAC.42.4.849.
13. Lugo, G.; Castañeda-Hernández, G. Amikacin bayesian forecasting in critically ill patients with sepsis and cirrhosis. *Ther. Drug Monit.* **1997**, *19*, 271–276, doi:10.1097/00007691-199706000-00005.
14. Lugo, G.; Castañeda-Hernández, G. Relationship between hemodynamic and vital support measures and pharmacokinetic variability of amikacin in critically ill patients with sepsis. *Crit. Care Med.* **1997**, *25*, 806–811, doi:10.1097/00003246-199705000-00016.
15. Debord, J.; Pessis, C.; Voultoury, J.C.; Marquet, P.; Lotfi, H.; Merle, L.; Lachâtre, G. Population pharmacokinetics of amikacin in intensive care unit patients studied by NPEM algorithm. *Fundam. Clin. Pharmacol.* **1995**, *9*, 57–61, doi:10.1111/j.1472-8206.1995.tb00266.x.
16. Maire, P.; Barbaut, X.; Girard, P.; Mallet, A.; Jelliffe, R.W.; Berod, T. Preliminary results of three methods for population pharmacokinetic analysis (NONMEM, NPML, NPEM) of amikacin in geriatric and general medicine patients. *Int. J. Biomed. Comput.* **1994**, *36*, 139–141, doi:10.1016/0020-7101(94)90106-6.
17. Debord, J.; Voultoury, J.C.; Lachatre, G.; Gay, C.; Favereau, J.P.; Gay, R. Population pharmacokinetic parameters for bayesian monitoring of amikacin therapy in intensive care unit patients. *Eur. J. Clin. Pharmacol.* **1992**, *43*, 435–436, doi:10.1007/BF02220623.
18. Jelliffe, R.W. Creatinine clearance: Bedside estimate. *Ann. Intern. Med.* **1973**, *79*, 604–605, doi:10.7326/0003-4819-79-4-604.
19. Levey, A.S.; Stevens, L.A.; Schmid, C.H.; Zhang, Y.L.; Castro, A.F. 3rd; Feldman, H.I.; Kusek, J.W.; Eggers, P.; Van Lente, F.; Greene, T.; et al. A new equation to estimate glomerular filtration rate. *Ann. Intern. Med.* **2009**, *150*, 604–612, doi:10.7326/0003-4819-150-9-200905050-00006.
20. Robert, S.; Zarowitz, B.J.; Peterson, E.L.; Dumler, F. Predictability of creatinine clearance estimates in critically ill patients. *Crit. Care Med.* **1993**, *21*, 1487–1495, doi:10.1097/00003246-199310000-00016Robert, S.; Zarowitz, B.J.; Peterson, E.L.; Dumler, F. Predictability of creatinine clearance estimates in critically ill patients. *Crit. Care Med.* **1993**, *21*, 1487–1495, doi:10.1097/00003246-199310000-00016.
21. Cockcroft, D.W.; Gault, H. Prediction of creatinine clearance from serum creatinine. *Nephron* **1976**, *16*, 31–41, doi:10.1159/000180580.
